# Access to Street Markets and Consumption of Fruits and Vegetables by Adolescents Living in São Paulo, Brazil

**DOI:** 10.3390/ijerph15030517

**Published:** 2018-03-14

**Authors:** Luana Romão Nogueira, Mariane de Mello Fontanelli, Breno Souza de Aguiar, Marcelo Antunes Failla, Alex Antonio Florindo, Ligia Vizeu Barrozo, Moisés Goldbaum, Chester Luiz Galvão Cesar, Maria Cecilia Goi Porto Alves, Regina Mara Fisberg

**Affiliations:** 1 Departamento de Nutrição, Faculdade de Saúde Pública, Universidade de São Paulo, São Paulo, SP 01246-904, Brazil; luanaromaon@hotmail.com (L.R.N.); marianefontanelli@gmail.com (M.d.M.F.); 2 Coordenação de Epidemiologia e Informação, Prefeitura de São Paulo, São Paulo, SP 01223-010, Brazil; bsaguiar@prefeitura.sp.gov.br (B.S.d.A.); marcelofailla@prefeitura.sp.gov.br (M.A.F.); 3 Escola de Artes, Ciências e Humanidades, Universidade de São Paulo, São Paulo, SP 03828-000, Brazil; aflorind@usp.br; 4 Departamento de Geografia da Faculdade de Filosofia, Letras e Ciências Humanas, Universidade de São Paulo, São Paulo, SP 05508-080, Brazil; lija@usp.br; 5 Departamento de Medicina Preventiva, Faculdade de Medicina, Universidade de São Paulo, São Paulo, SP 01246-903, Brazil; mgoldbau@usp.br; 6 Departamento de Epidemiologia, Faculdade de Saúde Pública, Universidade de São Paulo, São Paulo, SP 01246-904, Brazil; clcesar@usp.br; 7 Departamento de Saúde do Estado de São Paulo, Instituto de Saúde, São Paulo, SP 01314-000, Brazil; ceciliagoi2@gmail.com

**Keywords:** food environment, fruits, vegetables, street markets, adolescents

## Abstract

Food environment and income act as determinants of diet, and consequently, of the consumption of fruits and vegetables. The objective of this study is to investigate the association between fruit and vegetable consumption, income, and street market density in adolescents living in São Paulo, Brazil. Data from 521 adolescents (12 to 19 years) participating in the 2015 Health Survey of São Paulo were used. Buffers (500, 1000, and 1500 m) were drawn around the households and the street markets were counted in each zone. Multilevel logistic regression models were used to evaluate the association between fruit and vegetable consumption, income, and street market density. The main results showed that the presence of a street market in the zone closest to the households (500 m) was associated with higher consumption of fruits and vegetables (OR: 1.73; CI 95% 1.01–3.00). Higher family income was associated with a higher consumption of fruits and vegetables for models of 500 m buffer (OR: 2.56; CI 95% 1.47–4.45), 1000 m (OR: 2.30; CI 95% 1.33–3.96), and 1500 m (OR: 2.32; CI 95% 1.35–4.00). These results support the implementation of public policies that jointly consider income and the availability of street markets or healthy food environments.

## 1. Introduction

Fruits and vegetables (FV) are important components of a healthy diet because they are sources of fiber, vegetable proteins, and protective micronutrients. Therefore, since 2004, the World Health Organization (WHO) has emphasized the need for initiatives to make these foods more accessible [1]. A study conducted with data from 1990 to 2013 from 188 countries with the aim of quantifying contributing risk factors for the disability-adjusted life-years (DALYs) identified that among dietary factors, low consumption of FV are the main contributors to the DALYs, occupying the first and fourth position, respectively. Together, the low consumption of FV was responsible for about 5 million deaths and more than 113 million DALYs in 2013 [2]. A critical review reinforces the importance of FV consumption, especially due to the prevention of cardiometabolic diseases, such as cardiovascular diseases and hypertension [3].

The intake of FV can be considered more decisive during adolescence, when there is a greater need for nutrients, because it is a period of growth, development, and the establishment of habits that tend to remain in adult life [4,5]. Despite this, Albani et al. [6], who evaluated FV consumption between the ages of 2 and 23 after four waves of a British cohort, found a decrease in FV consumption from the age of 7, reaching its lowest level during adolescence. For Brazilian adolescents, the prevalence of fruit consumption is also low, as it was among the 20 foods most consumed only in the group of boys 12 to 13 years old [7]. Corroborating these results, Andrade et al. [8] compared the diet quality of the years 2003 and 2008 in a population-based study in adolescents, adults, and elderly people living in São Paulo, and observed a reduction in the Revised Brazilian Health Eating Index for adolescents, also influenced by the decrease of the scores for components related to FV consumption.

Initiatives to promote FV consumption generally focus only on the individual level; however, studies have shown that the food environment, defined as a collective physical, economic, political, and sociocultural environment that provides opportunities and conditions that influence food and beverage choices can be a determinant of diet, influencing FV intake [9,10,11,12]. Individuals’ personal characteristics interact with the food environment to mold their diet [13,14]. For this reason, studies have investigated the role of the environment in FV consumption, specifically in adolescents, be it in the domestic, school, or community environment [15,16,17,18]. However, these studies were conducted in the United States, which is a high-income country.

Among an individual’s personal characteristics, income plays an important role in FV consumption, as it reflects the availability of resources that make healthy and nutritious food more accessible [19]. In this context, a review that investigated studies that related food prices, diet quality, and socioeconomic status found that healthy diets are costlier, so that cheaper and energy-dense diets, in most cases with FV deficiencies, tend to be selected in several different countries by low-income groups [20]. Previous studies have shown that income influences FV consumption, especially in adolescents, who consume smaller amounts of these foods [19,21,22].

Considering the paucity of evidence of the role of the environment in diet in middle-income countries, the objective of this study is to investigate the association between FV consumption, income, and street market density among adolescents living in São Paulo city, an important megacity and economic center of Brazil that has undergone extensive urban expansion in the last century.

## 2. Materials and Methods

### 2.1. Study Sample

Data from the 2015 Health Survey of São Paulo (2015 ISA-Capital) were used. This is a cross-sectional, population-based study with a probabilistic sample of individuals living in permanent households located in the urban area of São Paulo city, Brazil.

The sample was obtained using stratified sampling by clusters carried out in two stages: census tracts and domicile. The census tracts were stratified in the five health administrative areas of São Paulo city (north, mid-west, southeast, south, and east). For the first stage, within each coordination, 30 census tracts were selected. For the second stage, an average of 18 households were randomly selected by census tract. In the households selected, all the people belonging to the domains of interest were interviewed. In order to make up for sample losses, more independent draws were made, resulting in a sample that allows estimates of proportions of 0.50, with a sampling error of seven percentage points, considering a 95% confidence level and a delineation effect of 1.5.

In 2015 ISA-Capital, the following inclusion criteria were considered: age greater than 12 years, of both sexes; living in the urban area of São Paulo city during the period of the survey; and, in the case of women, not being pregnant or lactating during data collection.

At 2015 ISA-Capital, 4059 interviews were conducted. For nutritional information collection, a subsample was calculated (ISA-Nutrition), whereby 1737 individuals were randomly selected to perform the first 24 h dietary recall (24HR) and participate in other phases of the study. Further details on the ISA-Nutrition procedures can be found elsewhere [23].

For this study, only adolescent data (12 to 19 years old) in the sample were used. Of the 553 adolescents who participated in ISA-Nutrition, 521 were included in the present study because they answered the structured questionnaire, at least one 24HR, and the geocoded residence.

2015 ISA-Capital was approved by the research ethics committees of the Faculty of Public Health and the Municipal Health Department (CAAE No. 32344014.3.0000.5421), as well as the present study (CAAE No. 65228317.1.0000.5421). Participation of the research subjects was conducted after they signed a written informed consent.

### 2.2. Data Collection and Processing

Demographic, socioeconomic, and lifestyle data were collected through a structured questionnaire validated in a pilot study. For the present study, the following variables were used: age (categorized from 12 to 15 years, and 16 to 19 years); sex (male and female); race (white and non-white, with black, indigenous, East Asian, and others considered as non-white); family income per capita (less than or equal to minimum wage, greater than minimum wage, and no response); education of the head of household (less than or equal to 9 years, 10 to 12 years, and greater than 12 years). Length of residence at the same residence was collected with the question, “How long have you lived in this same home?”. The variable years of residence was then categorized as up to five years or more than five years.

Information on physical activity practices was obtained with the International Physical Activity Questionnaire—IPAQ long version [24], which has been validated in Brazil [25]. Physical activity in leisure was categorized as follows: does not comply with the recommendation (<420 min/week), and complies with the recommendation (≥420 min/week) for adolescents of 17 years or less. For adolescents of ages 18 and 19, the following were used: does not comply with the recommendation (<150 min/week) and complies with the recommendation (≥150 min/week), according to World Health Organization guidelines [26].

Weight and height information were self-reported. Information on participants’ weights was collected by the question, “What’s your weight?”, whose response was given in kilograms or grams. Height was collected with the question “What’s your height?”, with response options in meters or centimeters. From these data, the body mass index (BMI) for each participant was calculated from the formula of weight in kilograms divided by the square of height in meters (kg/m^2^), in order to classify their nutritional status according to WHO standards [27]. Individuals were classified as non-overweight (percentile < 85) and overweight (percentile ≥ 85) [27]. The self-reported measure was validated in a previous study with the same population, which observed high sensitivity (91.67%) and specificity (97.67%) for this information [28].

The intramunicipal Human Development Index (HDI) was taken from the United Nations Development Programme Atlas for Brazil [29], which addresses three dimensions of the region: income, longevity, and education. The Brazilian intramunicipal HDI considers the same three dimensions as the Global HDI with adaptations of the global methodology to the Brazilian context, and taking into account the availability of national indicators.

### 2.3. Food Consumption Data

Food consumption was collected with two 24HRs within one year (February 2015 to February 2016). Data were collected on non-consecutive days, representing all days of the week and seasons. The first 24HR was collected during the first home visit through the multiple-pass method, a standardized process that aims to keep the individual interested and engaged in the interview while helping him to remember all the items consumed [30]. The second 24HR was collected from 291 adolescents, and was conducted by telephone about 173 days after the first 24HR collection using the automated multiple-pass method. Data were typed in the Nutrition Data System for Research (NDS-R) software, version 2014, developed by the Nutrition Coordinating Center, University of Minnesota, Minneapolis, MN, USA. This software uses the food composition table developed by the United States Department of Agriculture as a data source, therefore, the nutritional values of the foods present in the program were compared with the nutritional values of foods available in national tables [31].

### 2.4. Fruits and Vegetables

Usual FV consumption was estimated using the multiple source method [32], an online platform that estimates habitual intake of nutrients and foods based on data from two or more short-term food surveys (such as 24HR) collected for the whole sample or for a part of it. The adequacy of consumption was evaluated according to WHO recommendations [1], which recommend the consumption of at least 400 grams per day of FV. The usual intake in grams per day was categorized as less than 75th percentile (<P75) or greater than or equal to the 75th percentile (≥P75).

### 2.5. Street Markets and Households

Geocoding of adolescent residence addresses was performed in Google Earth Pro version 7.1 (Mountain View, CA, USA) using the geographic coordinate system with WGS 84 datum. Of the 553 adolescents interviewed, residence addresses of 521 (94.2%) were geocoded. Geocoded records were validated by geographic operation in the QGIS program version 2.16.1 (Development Team, 2016), based on the overlapping of the geographic coordinates obtained with the census tracts selected in the study.

Street market locations in the city of São Paulo were obtained from São Paulo Secretariat of Urbanism and Licensing updated in 2015, available on the website Geosampa [33]. In order to verify the presence of these establishments in the surroundings of the adolescents’ households, areas of influence (buffers) with radii of 500, 1000, and 1500 m were created in the program QGIS version 2.16.1 (Development Team, 2016) and the density of the establishments in each one of these areas was counted.

### 2.6. Statistical Analysis

Socioeconomic and lifestyle variables were described using absolute and relative frequencies. Differences between the variables by FV consumption (<P75 and ≥P75) were tested according to Pearson’s chi-square test. The distribution of adolescents according to street market density in the buffers was described by means of absolute and relative frequencies.

Data were analyzed with Stata software version 13.0 (StataCorp, College Station, TX, USA) using multilevel logistic regression models. The dependent variable was FV consumption in grams, categorized as <P75 or ≥P75. The independent variables were street market densities in the buffers of 500, 1000, and 1500 m, and the family income per capita. Models were adjusted for age, sex, BMI, years of residence, health administrative areas, and HDI intramunicipal. Results were presented as odds ratios (OR) and 95% confidence intervals (95% CI).

## 3. Results

**Figure 1:**
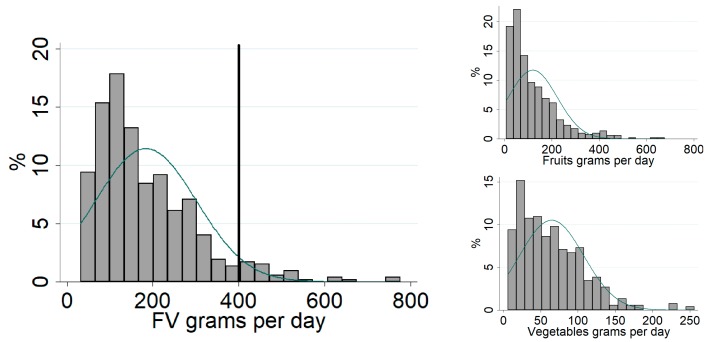
Distribution of usual consumption in grams of fruits and vegetables by adolescents. ISA-Capital, São Paulo city, Brazil, 2015. Source: 2015 ISA-Capital.

Table 1 contains the descriptive analysis of the sample according to FV consumption. The mean age of adolescents was 15.5 (SD 2.29) years, 264 (50.7%) of whom were male, 296 (57.3%) non-white, and 296 (59.6%) had income per capita family less than or equal to a minimum wage, and 242 (48.9%) had a household head with 9 years of education or less. Regarding leisure time physical activity, 418 (80.2%) did not meet the WHO minimum recommendation, and 355 (70.4%) were not overweight. There was a statistically significant difference between the categories of FV consumption by income, household head education, and leisure time physical activity. Only 32 (6.1%) adolescents reached the WHO recommendation of daily consumption of at least 400 grams of FV (Figure 1).

**Figure 2:**
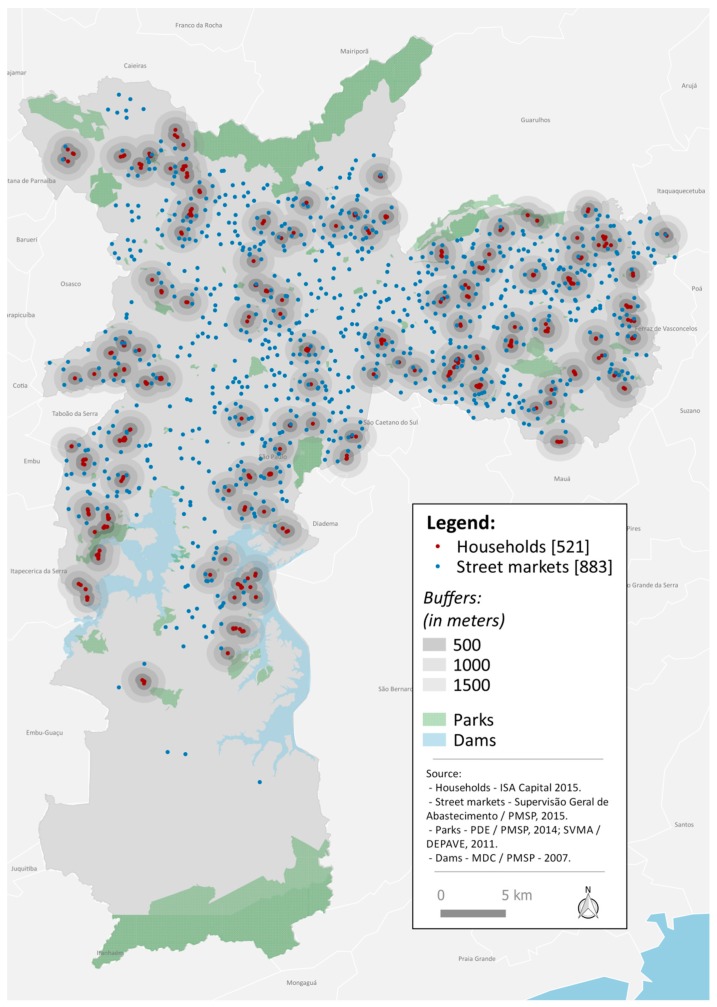
Spatial distribution of street markets in the São Paulo city and adolescent households. ISA-Capital, São Paulo, Brazil, 2015.

The distributions of the adolescents who participated in the study and of the 883 street markets in the city of São Paulo are shown in Figure 2. Street market density varied according to the size of the buffer (Table 1). In the 500 m buffer, 49.5% of adolescents did not have street markets around their residence, while for the 1000 m buffer, 53.9% had from 2 to 4 street markets near the residence, and for the 1500 m buffer, 44.5% had from 3 to 7 street markets near the residence.

For the multilevel logistic regression models, we used the cutoff point of P75, which corresponds to 243.3 grams. We chose this cutoff point since the median ingestion was low (151.3 grams). Turning to the results of the adjusted models, having a street market in the 500 m buffer was positively associated with higher FV consumption. This association was found regardless of sex, age, BMI, years of residence, region of residence in São Paulo, and HDI intramunicipal (Table 2). No associations were found between street market density and FV consumption for the buffers of 1000 and 1500 m.

For all three buffers sizes (500, 1000, and 1500 m), family income per capita greater than a minimum wage was positively associated with higher FV consumption, regardless of sex, age, BMI, years of residence, region of residence in São Paulo, and HDI intramunicipal (Table 2).

## 4. Discussion

The present study showed low FV consumption among adolescents; however, the presence of a street market near the adolescent’s household (500 m buffer) was associated with higher consumption of these foods in this population. No associations were found for higher street market density in the evaluated buffers, highlighting the importance of proximity to household and income, and suggesting that higher density might not be relevant to increased consumption of these foods in this population.

These findings corroborate other studies that also highlight the importance to the use of public spaces and food stores of proximity to the home [13,34]. In a study by Duran et al. [35] with 1842 individuals residing in São Paulo, lower FV consumption was found for those individuals who lived further from the supermarket or street market. In addition, an association was also found for FV consumption and the density of these establishments in the 1600 m buffers. The different results in relation to density are largely due to the age group of the study population, since Duran et al. included only adults. There are also differences in the measurement of FV consumption, as in that study it was measured through questions on the usual consumption of these foods during the week. In this study, we evaluated FV consumption adequacy using WHO recommendations, which facilitates comparison with international studies. Although national recommendations encourage FV consumption, there is no cutoff point for assessing adequacy of consumption. The latest quantitative recommendation for the Brazilian population was based on the WHO recommendation [36].

Street markets in Brazil are weekly markets characterized by the sale of mostly fresh foods with minimum degree of processing [35]. They are regulated by the City Hall and have a commercial character, but also promote popular culture and the sale of healthy food with greater diversity and lower prices, as these are important public tools of the FV trade [37,38]. A study that evaluated the FV availability and diversity in the city of São Paulo concluded that street markets are less vulnerable to seasonality. Although the study did not evaluate differences between regions of the city (city center and districts outside the city center), street markets presented greater diversity and lower prices when compared to supermarkets in regions with the same HDI [39]. However, evidence shows that adolescents are more willing to buy processed foods than FV [40,41,42], which suggests less use of these spaces by this group. In a review of the literature by Rasmussem et al. [43], some more consistent determinants of FV consumption by children and adolescents are preference, parental consumption, and residential availability. Thus, while adolescents may not use the street markets, they can benefit if their parents use them. Brazilian national policies reinforce the need to create favorable environments for health, including social participation to improve local food environments [44,45]. In addition, the Food and Nutrition Education Reference Framework for public policies (Marco de Referência de Educação Alimentar e Nutricional para as Políticas Públicas) refers to street markets as an important public area of food and nutritional education [46]. 

Several studies show income to be a relevant factor in FV consumption [47,48,49]. Despite the lower price of food in street markets, studies describe the relationship between higher income and healthy diet, including FV consumption. Higher costs may explain socioeconomic disparities in diet quality [20]. Claro and Monteiro [47], when analyzing the influence of income and food prices on FV purchased by Brazilians, found using data on household availability that lower FV prices and increases in monthly income per capita were associated with an increase in the participation of these foods in Brazilian diets. John and Ziebland [50], conducting a qualitative study to verify the barriers to FV consumption, found that high costs and the inaccessibility of places to purchase them remain relevant barriers to FV purchase. That is, the proximity and greater density of places to buy healthy foods do not, by themselves, reduce the inequality in access to these foods, which reveals the need to implement public policies that act on these variables together [48].

Some limitations of the study should be considered. First, most of the variables are based on self-reported data, such as the majority of nutritional epidemiology studies. Second, we only use households as reference points to determine access to street markets; however, using the residence alone does not fully reflect the environment to which the individual is exposed. Work and school environment also play an important role in food choices. Considering that 76.2% of the sample participants were students, and 22.1% of them worked (results not shown), the inclusion of variables related to their school and work environments could increase our understanding of their FV consumption. Similarly, the buffers that were used in the study may not represent the areas where individuals actually purchase food. However, this method is one of those most used in studies with this theme, since administrative boundaries do not restrict daily life [35]. In addition, using different buffer sizes allows us to investigate the complex relationships and interactions between individuals and their environment, since there is no consensus on buffer sizes in studies of food environment variables [51,52]. Although, the lack of direct measurement of the FV purchase in street markets is also a limitation; however, official data regarding the street markets made available by the Municipality of São Paulo (GeoSampa) were used [33]. Estimates of FV consumption were assessed using two 24HR with standardized methods that reduce memory bias and adjust for intrapersonal variability [32]. Another strength of this study is the adjustment for intramunicipal HDI, since this classification allowed us to gather locations with similarities in income, longevity, and education. In addition, a previous study found that FV prices are higher in districts with high HDI when compared to others [39].

Despite the limitations, because the present study used data from a population-based study conducted in a megacity with more than 12 million inhabitants that has unique urban dynamics conditions, it was able to contribute to the understanding of the role of the environment in FV consumption among the adolescents of a middle-income country, where studies of this subject are still scarce.

## 5. Conclusions

The presence of a street market near adolescents’ households (within a 500 m buffer) was associated with higher FV consumption. Independent of the buffer size and street market density, family income per capita greater than the minimum wage was positively associated with higher FV consumption. Thus, joint consideration of income and availability of street markets in the implementation of public policies may be an alternative for increasing FV consumption among adolescents living in São Paulo, Brazil.

## Figures and Tables

**Table 1:** Demographic, socioeconomic, lifestyle factors, and street markets density by fruits and vegetables consumption of adolescents living in the city of São Paulo, Brazil, 2015.

Variables	*n* (%)	<P75	≥P75	*p* Value
		*n* (%)	*n* (%)	
Age (years, *n* = 521)				
12–15	270 (51.8)	208 (77.1)	62 (22.9)	0.227
16–19	251 (48.2)	183 (72.9)	68 (27.1)	
Sex (*n* = 521)				
Male	264 (50.7)	197 (74.6)	67 (25.4)	0.820
Female	257 (49.3)	194 (75.4)	63 (24.6)	
Race (*n* = 517)				
White	221 (42.7)	158 (71.5)	63 (28.5)	0.088
Non-white	296 (57.3)	231 (78.0)	65 (22.0)	
Family income per capita (*n* = 497) *				
≤1 Minimum wage	296 (59.6)	235 (79.4)	61 (20.6)	0.039
>1 Minimum wage	121 (24.3)	82 (67.8)	39 (32.2)	
No response	80 (16.1)			
Education of the household head (*n* = 495)				
≤9 years	242 (48.9)	194 (80.2)	48 (19.8)	0.003
10–12 years	157 (31.7)	103 (65.6)	54 (34.4)	
>12 years	96 (19.4)	76 (79.2)	20 (20.8)	
Leisure physical activity (*n* = 521)				
Does not comply with recommendation	418 (80.2)	323 (77.3)	95 (22.7)	0.018
Complies with recommendation	103 (19.8)	68 (66.0)	35 (34.0)	
Body Mass Index (*n* = 504)				
Non-overweight	355 (70.4)	266 (74.9)	89 (25.1)	0.707
Overweight	149 (29.6)	114 (76.5)	35 (23.5)	
Years of residence (*n* = 521)				
≤5	184 (35.3)	134 (72.8)	50 (27.2)	0.386
>5	337 (64.7)	257 (76.3)	80 (23.7)	
Street market density 500 m buffer				
0	258 (49.5)	198 (76.7)	60 (23.3)	0.055
1	155 (29.8)	106 (68.4)	49 (31.6)	
≥2	108 (20.7)	87 (80.6)	21 (19.4)	
Street market density 1000 m buffer				
≤1	124 (23.8)	98 (79.0)	26 (21.0)	0.268
2–4	281 (53.9)	203 (72.2)	78 (27.8)	
≥5	116 (22.3)	90 (77.6)	26 (22.4)	
Street market density 1500 m buffer				
≤2	101 (19.4)	84 (83.2)	17 (16.8)	0.093
3–7	232 (44.5)	167 (72.0)	65 (28.0)	
≥8	188 (36.1)	140 (74.5)	48 (25.5)	

Source: ISA-Capital 2015. * Minimum wage value in 2015 was 224.02 USD.

**Table 2:** Odds ratios for associations between fruits and vegetables consumption, income, and street market density in different buffers sizes. ISA-Capital, city of São Paulo, 2015.

Variable	OR (95% CI)
500 m buffer	
Street market density	
0	ref
1	1.73 (1.01–3.00) *
≥2	0.70 (0.35–1.42)
Family income per capita	
≤1 Minimum wage	ref
>1 Minimum wage	2.56 (1.47–4.45) *
1000 m buffer	
Street market density	
≤1	ref
2–4	1.33 (0.70–2.53)
≥5	0.93 (0.41–2.12)
Family income per capita	
≤1 Minimum wage	ref
>1 Minimum wage	2.30 (1.33–3.96) *
1500 m buffer	
Street market density	
≤2	ref
3–7	1.97 (0.96–4.04)
≥8	1.51 (0.67–3.44)
Family income per capita	
≤1 Minimum wage	ref
>1 Minimum wage	2.32 (1.35–4.00) *

Models adjusted for age, sex, body mass index (BMI), years of residence, human development index (HDI) intramunicipal and region of São Paulo; * *p* < 0.05. Ref: reference category.
